# Psychosocial Correlates of Food Addiction and Its Association with Quality of Life in a Non-Clinical Adolescent Sample

**DOI:** 10.3390/nu10070837

**Published:** 2018-06-28

**Authors:** Zhongyi Zhao, Yanan Ma, Yanshuo Han, Yang Liu, Keming Yang, Shihan Zhen, Deliang Wen

**Affiliations:** 1 School of Public Health, China Medical University, Shenyang 110122, China; zyzhao@cmu.edu.cn (Z.Z.); ynma@cmu.edu.cn (Y.M.); yanshuohan@cmu.edu.cn (Y.H.); yliu0568@cmu.edu.cn (Y.L.); shzhen@cmu.edu.cn (S.Z.); 2 Richard M. Fairbanks School of Public Health, Indiana University, Indianapolis, IN 46202, USA; kemyang@iu.edu

**Keywords:** adolescent, food addiction, psychosocial factors, quality of life

## Abstract

Background: Most studies related to food addiction have focused on assessing food addiction among adult populations. However, evidence in adolescents has been limited. The aim of this study was to investigate the prevalence of food addiction in a non-clinical adolescent sample. Psychosocial correlations of food addiction and associations with different quality of life dimensions were also tested. Methods: The sample included 593 Chinese adolescents (51.9% female; age range: 13–17 years). All participants provided sociodemographic information and completed questionnaires regarding food addiction, depression, self-esteem, loneliness, psychosocial problems, and quality of life (QoL). Results: The prevalence of food addiction was 6.91% in our sample. A multivariable logistic regression indicated that food addiction was associated with depression (AOR = 2.58; 95% CI: 1.32–5.05), low self-esteem (AOR = 2.75; 95% CI: 1.31–5.78), and loneliness (AOR = 2.30; 95% CI: 1.14–4.65). After multivariable adjustments for sociodemographic and psychological variables, food addiction was associated with lower overall QoL and psychosocial health of QoL. Conclusions: Food addiction may be common among Chinese adolescents. Food addiction was associated with depression, low self-esteem, and loneliness. Lastly, food addiction was also associated with lower overall QoL and psychosocial health of QoL. Future public health programs and interventions consider targeting the factors associated with food addiction to increase healthy eating behaviors among adolescents.

## 1. Introduction

Similar to addiction triggered by traditional addictive substances (e.g., tobacco, alcohol, and cocaine), food addiction may be related to the addictive and rewarding effects of highly processed foods (e.g., foods with high levels of sugar, fat, and salt) [1]. Individuals who exhibit dietary patterns similar to the typical behavior of people addicted to drugs are described as food addicts. Currently, the Yale Food Addiction Scale (YFAS) is the only psychometric assessment tool available to measure the construct of food addiction [2]. A recent meta-analysis found that the weighted mean prevalence of food addiction based on assessments using different versions of the YFAS was 19.9% in clinical and non-clinical samples [3]. However, most studies published so far have focused on assessing food addiction among adult populations. Evidence in adolescents has been limited.

In recent years, researchers have emphasized the importance of understanding food addiction among adolescents [4]. Adolescents view rewarding and aversive stimuli differently from adults by typically showing a shift toward enhanced sensitivity to rewards but attenuated aversive sensitivities with regard to addictive substances [5]. A longitudinal study found that the reward of sweet stimuli was significantly higher in individuals aged 11–15 years than in adults [6]. These differences indicate that adolescent populations may be particularly prone to developing food addiction when they come in contact with high-sugar or high-calorie foods. Furthermore, compared with adults, adolescents have exhibited more characteristics associated with an increase in substance use such as impulsiveness, sensation seeking, and risk taking [7]. These features make adolescents more susceptible to developing addictive-like eating behaviors. To date, studies on food addiction in adolescents have been scarce with the reported prevalence in these studies ranging from 2.6% to 38% [8,9,10,11,12,13]. For instance, Meule et al. (2015) reported a prevalence of food addiction of up to 38% in a sample of 50 overweight and obese German adolescents who sought treatment at a weight-loss hospital [9] while another study examining the prevalence of food addiction in a large sample of Dutch adolescents (*N* = 2653) aged 14–21 years observed a lower prevalence of 2.6% [8].

Many studies have reported the associations between food addiction and a number of psychosocial correlations in adults [14]. However, such associations in adolescent populations are poorly understood and inconsistent results have been reported by a limited number of epidemiological studies. For example, at present, the association between food addiction and depression in adolescents is unclear. A previous study found that overweight and obese adolescents with food addiction exhibited more symptoms of depression [9]. However, in another study, it was observed that patients with food addiction did not report more symptoms of depression [11]. Self-esteem is closely associated with eating disorders and low self-esteem has been regarded as a key risk factor for eating disorders [15]. Current evidence shows that food addiction is associated with low self-esteem in obese adults with a binge eating disorder [16]. However, this association has not yet been examined in adolescents. Another factor that might be related to food addiction is loneliness. Existing evidence revealed the relationship between loneliness and disordered-eating behavior [17]. Loneliness can directly increase binge eating and increase the risk of obesity. Characteristics related to loneliness are significantly associated with eating disorders such as emotional eating and binge eating disorder. Therefore, an understanding of the effect of loneliness on food addiction is important for prevention strategies. A recent study has shown that there is a significant association between food addiction and loneliness among undergraduate students [18]. However, no studies have investigated the association between food addiction and loneliness in adolescents.

Recently, researchers have found that food addiction is associated with a poorer quality of life (QoL). A largescale, web-based epidemiologic study indicated that food addiction was independently associated with reductions across all QoL dimensions [19]. Another study showed that food addiction was associated with poorer QoL in obese adolescents seeking weight loss [12]. However, no studies have reported the association of food addiction with different QoL dimensions in non-clinical adolescent samples.

In view of the differences in the aforementioned literature, the present study had three objectives: (1) to investigate the prevalence of food addiction in a non-clinical sample of Chinese adolescents; (2) to investigate the correlations of food addiction with psychosocial factors such as depression, self-esteem, and loneliness; and (3) to investigate the independent associations of food addiction with different QoL dimensions. We hypothesized that food addiction would be prevalent in the non-clinical adolescent sample of the study but that the prevalence would be lower than the weight-loss-seeking clinical population and is significantly associated with psychosocial factors including depression, self-esteem, and loneliness. In addition, we hypothesized that adolescents with food addiction would have lower QoL compared with peers without food addiction after adjusting for common sociodemographic and psychopathological confounders.

## 2. Methods

### 2.1. Participants

Students in grades 7–11 from three public middle schools in Shenyang, China participated in our study from October to December 2017. First, the investigator informed students in advance of the purpose and methods of the study and their right to decline participation. Then all participating students in the classroom were invited to complete a paper version of the questionnaire, which include sociodemographic characteristics and self-report scales under the supervision of a teacher and an investigator of the research team. Anthropometric data collections were conducted within one week after the questionnaire was completed. Completed questionnaires were received from 608 students with a participation rate of 97.1%. However, those who returned questionnaires with missing or incomplete YFAS data (*N* = 15) were excluded from the study. Ultimately, 593 (51.9% female, *N* = 308) valid questionnaires were included in the statistical analysis. Written informed consent was obtained from all participants and their parents prior to the study. Ethical approval for the study was granted by the ethics committee at China Medical University.

### 2.2. Measures

#### 2.2.1. Yale Food Addiction Scale (YFAS)

The presence of food addiction was assessed using the Chinese version of the YFAS [10]. This self-report scale includes 25 items and was developed based on the diagnostic criteria for substance dependence in the Diagnostic and Statistical Manual of Mental Disorders, Fourth Edition, Text Revision (DSM-IV-TR) [20] to identify individuals potentially addicted to highly processed foods [2]. Participants who satisfied three or more of the seven diagnostic criteria as well as the criteria for clinically significant impairment or distress received a “diagnosis” of food addiction. It is worth noting that the “diagnosis” of food addiction based on YFAS measurement has the same sense as that of substance dependence from DSM-IV-TR and does not represent clinical diagnosis. The Chinese version of the YFAS has already been validated in Chinese female adolescents [10]. The factor structure of the YFAS in Chinese male adolescents was further validated by our research team and the single-factor model showed an acceptable fit (CFI = 0.908, RMSEA = 0.057).

#### 2.2.2. Center for Epidemiology Scale for Depression (CES-D)

The Chinese version of the CES-D, which was previously validated [21] and is widely used in studies on Chinese adolescents [22], was used to determine if individuals had symptoms of depression. Participants were asked to indicate the frequency of each depressive symptom experienced in the past week. The total score ranges from 0 to 60 with higher scores indicating higher levels of depression [23] and a score of 16 represents the optimal cutoff point for identifying major depressive disorder [24].

#### 2.2.3. Rosenberg Self-Esteem Scale (RSES)

The RSES is one of the most widely used measures for assessing global self-esteem [25]. This self-report scale comprises 10 items, which are answered on a four-point Likert scale (0–3 points). The total score ranges from 0 to 30 with higher scores indicating higher self-esteem and scores lower than 15 indicating low self-esteem [26]. The Chinese version of the RSES is widely used in studies on Chinese adolescents [27].

#### 2.2.4. UCLA Loneliness Scale

The UCLA Loneliness Scale is the most widely used measure for assessing loneliness [28] and has been applied to various populations including adolescents [29]. This scale consists of 20 items rated on a four-point Likert scale (1–4 points), and the total score ranges from 20 to 80 with higher scores indicating stronger subjective feelings of loneliness. Although a cutoff score was not defined in the original UCLA Loneliness Scale, based on previous literature, the cutoff score for loneliness is typically one standard deviation above the mean sample score [30]. In our study, the mean loneliness score and standard deviation of the sample were 33.8 and 11.1, respectively. Therefore, the cutoff score was calculated as 44.9. The Chinese version of the UCLA Loneliness Scale has already been used in many studies and proven to be valid and reliable [31].

#### 2.2.5. Strengths and Difficulties Questionnaire (SDQ)

The SDQ is a screening questionnaire used for identifying children and adolescents with a high risk for psychosocial problems [32]. In the present study, the self-report Chinese version of the SDQ, which has been validated in Chinese studies [33], was used to assess the level of psychosocial problems among the participants. The SDQ comprises 25 items, which are divided into five subscales including emotional symptoms, conduct problems, hyperactivity/inattention, peer relationship problems, and prosocial behavior. The scores of four of the subscales (excluding the prosocial behavior subscale) are added to obtain a total difficulties score, which ranges from 0 to 40 with high scores indicating more psychosocial problems.

#### 2.2.6. Pediatric Quality of Life Inventory™ Version 4.0 (PedsQL™ 4.0) Generic Core Scales

In the present study, the licensed self-report version of the PedsQL^TM^ 4.0 Generic Core Scales (https://eprovide.mapi-trust.org; www.pedsql.org) for children aged 13–18 was used to measure the different dimensions of health-related QoL of the participants [34,35]. The 23-item PedsQL™ 4.0 Generic Core Scales consists of four dimensions including physical functioning (eight items), emotional functioning (five items), social functioning (five items), and school functioning (five items). In particular, the score for the physical functioning dimension represents the physical health summary score (8 items) while the psychosocial health summary score (15 items) is computed from the scores from the emotional, social, and school functioning dimensions. Items are scored on a five-point Likert scale (0–4 points) where 0 = never, 1 = almost never, 2 = sometimes, 3 = often, and 4 = almost always. The items are then reverse-scored and linearly transformed to a 0–100 scale (0 = 100, 1 = 75, 2 = 50, 3 = 25, 4 = 0). To calculate the mean total scale score and mean score for each dimension, the sum of scores for the items are divided by the number of scored items with higher mean scores, which indicates better QoL. Previous studies have demonstrated that the Chinese version of the PedsQL™ 4.0 Generic Core Scales has satisfactory reliability and validity [36].

#### 2.2.7. Body Mass Index (BMI)

All anthropometric measurements were performed by the same researcher based on standard techniques recommended by the World Health Organization. Height was measured using a portable stadiometer (SECA 213, Hamburg, Germany) that was accurate to 0.1 cm. Body weight was measured using a body fat analyzer (TANITA DC430MA, Tokyo, Japan) that was accurate to 0.1 kg. BMI was calculated by dividing the participants’ body weight by the square of their height (kg/m^2^) and BMI z-scores were calculated.

#### 2.2.8. Sociodemographic Variables

The following information was collected from the participants: age, sex, ethnicity, whether they were the only child in the family, family structure, parents’ educational levels, monthly household income, and history of alcohol intake.

### 2.3. Statistical Analysis

The proportion of missing data at the variable level was within 0.5–2.0% and missing data were handled using the expectation maximization algorithm. The convergence and dispersion trends for quantitative variables were expressed as mean ± standard deviation and qualitative variables were expressed as frequencies and percentages. The prevalence of food addiction was calculated and the sociodemographic and psychosocial variables were compared between adolescents with and without food addiction. Continuous variables were compared using the independent-samples *t* test. Categorical variables were compared using Pearson’s chi-square test or Fisher’s exact test, as appropriate. The correlations of food addiction (dependent variable) with depression, self-esteem, loneliness, and SDQ psychosocial problems were analyzed using simple and multivariable logistic regression. With regard to the correlations between food addiction and psychosocial problems, the scores of the respective SDQ subscales were regarded as continuous variables in the model input. Sociodemographic factors were adjusted as confounding variables in all multivariable models. The correlations between food addiction and the respective QoL dimensions (dependent variables) were analyzed with multivariable regression models by adjusting for both sociodemographic and psychological factors. All statistical analyses were two-tailed with α = 0.05 as the significance level. SPSS 23.0 software for Windows was used.

## 3. Results

### 3.1. Participant Characteristics

General characteristics of the study sample were presented in Table 1. The mean age of participants was 15.0 ± 1.5 years (age range: 13–17 years) and the mean BMI was 19.6 ± 3.8 kg/m^2^. The majority of participants were Han Chinese (83.8%), were the only child of the family (75.7%), and belonged to a complete family (88.5%). In addition, a small minority of participants had a history of alcohol intake (2.0%). Among the participants, 27.8% had depressive symptoms, 15.2% had low self-esteem, and 19.2% suffered from loneliness.

### 3.2. Prevalence of Food Addiction

Among all adolescents in our sample, 6.91% satisfied the criteria for “diagnosis” with food addiction. Compared with participants without food addiction, adolescents with food addiction had a higher BMI (*p* = 0.016). No significant differences in sex, age, and other sociodemographic characteristics were found between the two groups (Table 1).

### 3.3. Psychosocial Correlation of Food Addiction

Results of simple and multivariable logistic regression analysis of food addiction and various psychosocial correlates were shown in Table 2. Simple logistic regression indicated that food addiction was positively correlated with depression, low self-esteem, and loneliness. The positive association between food addiction and depression (multivariable-adjusted Odds Ratio (AOR) = 2.58; 95% confidence interval (CI): 1.32–5.05), low self-esteem (AOR = 2.75; 95% CI: 1.31–5.78), and loneliness (AOR = 2.30; 95% CI: 1.14–4.65) remained significant after further adjustments for sociodemographic variables. Multivariable logistic regression models also showed that food addiction was significantly and positively associated with the SDQ psychosocial dimensions of emotional symptoms, conduct problems, hyperactivity/inattention, and peer relationship problems (All *p* < 0.05) but are not correlated with prosocial behavior (*p* > 0.05) (Table 2).

### 3.4. Associations of Food Addiction and QoL Dimensions

Compared to adolescents without food addiction, adolescents with food addiction had significantly lower PedsQL total scale scores, physical health and psychosocial health scores, and individual scores in the four QoL dimensions (physical functioning, emotional functioning, social functioning, and school functioning). The magnitude of the estimates regarding the inverse associations of food addiction with the various QoL dimensions remained basically unchanged after adjusting for sociodemographic variables. After multivariable adjustments for both sociodemographic and psychological variables, the inverse associations of food addiction with PedsQL total score, psychosocial health, emotional functioning, social functioning, and school functioning remained statistically significant while the association with physical health (physical functioning) became insignificant (Table 3).

## 4. Discussion

Although food addiction is still a controversial topic in the field of psychiatric medicine [37], it has received increasing interest in recent years [38]. The concept of food addiction has been developed by applying the DSM-IV-TR criteria for substance dependence to eating behaviors and were formalized in the Yale Food Addiction Scale [2]. Based on the Chinese version of YFAS, the present study revealed that the prevalence of food addiction in a non-clinical Chinese adolescent sample was 6.91%, which is comparable to the reported prevalence of 9.2% in Chinese female adolescents aged 14–19 years [10]. The prevalence of food addiction in adolescents varies across published studies conducted in different populations and settings (e.g., outpatients from an obesity clinic, inpatients, and general populations). For instance, Meule et al. (2015) [9] observed a food addiction prevalence of up to 38% in a sample of German adolescents seeking weight-loss treatment while Tompkins et al. (2017) [12] found that 30.7% of American adolescents who participated in a weight-management program satisfied the criteria for “diagnosis” with food addiction. In contrast, prevalence in general population samples of adolescents was lower with values of 2.6% [8] and 8.9% [39] reported in previous literature. Such differences in prevalence may be due to differences in BMI distribution between clinical and non-clinical study samples since more clinical samples of overweight and obese adolescents were included in the first few studies mentioned above [3].

In our study, the prevalence of food addiction did not differ by age and sex, which was consistent with previous studies [12]. In addition, in our general student samples, a significant and positive association of food addiction and BMI was observed. However, no association between food addiction and BMI was found in two previous studies conducted among patients seeking weight-loss treatment in clinical settings [9,12]. The results of the present study support the hypothesis proposed by Meule (2012) that a nonlinear relationship may exist between food addiction and BMI [40]. Specifically, although addictive eating patterns continuously exist in samples of severely obese individuals [9], the presence of a ceiling effect may restrict further increases in BMI when physical limits are reached. Therefore, the differences in BMI between patients with and without food addiction are frequently undetectable. Furthermore, in non-clinical samples that encompass a wider range of BMI, a positive correlation between food addiction and BMI may be observed more easily due to an increased severity of food addiction symptoms in overweight and moderately obese individuals. In addition, existing evidence suggests that eating-disordered behavior such as body dissatisfaction can serve as a mediating variable in the association between obesity and impairment in psychosocial functioning in adolescents [41]. This may also explain the observed association between food addiction and BMI.

In our study sample of 593 Chinese adolescents who were attending middle school, we observed that food addiction was associated with higher levels of depression. This is consistent with the results of a previous study [9], which also identified an association of food addiction with a higher number of depressive symptoms in obese adolescents seeking weight-loss treatment. However, in another study conducted in adolescent psychiatric inpatients, the reporting of a higher number of depressive symptoms by patients with food addiction was not observed as the high basal rate of symptoms in psychiatric disorders, which may have masked the relationship between the two variables [11]. We provide further epidemiological evidence for the association between food addiction and depression through a cross-sectional study in a population-based adolescent student sample. In addition, we also found that food addiction in adolescents was associated with lower self-esteem, which is consistent with results of studies conducted in adult samples [16,42]. To the best of our knowledge, this present study is the first study examining the relationship between food addiction and loneliness in adolescents. Our results showed high levels of loneliness are associated with addictive-like eating behaviors. This indicates that, when adolescents experience feelings of loneliness, they may direct their attention and project such feelings onto food—i.e., they may attempt to compensate for such feelings and eliminate loneliness through eating. In addition, it was observed in the present study that food addiction was associated with wider psychosocial problems such as emotional symptoms, conduct problems, hyperactivity/inattention, and peer relationship problems. In-depth investigation of the relationships between food addiction and these psychosocial variables in future studies will be useful in providing further evidence of the developmental factors that cause food addiction.

In the sample of the present study, after adjustments were made for sociodemographic and psychological variables, food addiction was associated with lower overall QoL and psychosocial health of QoL. This is also consistent with the results of a previous study [12], which indicated an association between food addiction and lower QoL in obese adolescents seeking weight loss. However, there was no control for psychological confounders. To the best of our knowledge, this is the first time that the associations of food addiction with various QoL dimensions have been investigated. Therefore, our data provide further epidemiologic evidence of the association between food addiction and QoL.

We acknowledge several limitations in this study. First, the study was conducted based on a convenience sample of students, which may not be representative of the entire Chinese adolescent population. Therefore, a selection bias may exist. It also suggests that estimates of the prevalence of food addiction derived from the current study in particular should be interpreted with caution. Second, variables of eating-disordered behaviors related to food addiction were not measured in the current study. This may lead us to be unable to determine whether and to what extent the associations between food addiction and psychosocial functioning might be due to the effects of eating-disordered behaviors. Future studies should simultaneously focus on food addiction and other eating-disordered behaviors, which will improve our ability to identify problematic eating behavior in health promotion and prevention programs. In addition, a self-report questionnaire was used in the study to assess food addiction and other psychosocial factors and correlations in adolescents. This could have resulted in a self-report bias due to the lack of clinical diagnoses of such factors. Furthermore, due to the development of YFAS 2.0 based on the recently published DSM, Fifth Edition (DSM-5), which has an increased number of addictive symptoms and lower threshold values for diagnosis, more participants may have been diagnosed with food addiction. In future studies, the measures used for assessing food addiction in adolescents must be updated to maintain consistency with the diagnostic criteria for substance dependence in the DSM-5. Lastly, since the present study was a cross-sectional study, this posed limitations on causal inferences involving correlations in food addiction.

## 5. Conclusions

In conclusion, in our sample of 593 Chinese adolescents aged 13–17 years who were attending middle school, we found that 6.91% of participants satisfied the diagnostic criteria for food addiction. Results of the present study also indicated that food addiction was associated with higher levels of depression, lower self-esteem, and stronger feelings of loneliness. Lastly, food addiction in adolescents was found to be closely associated with lower overall QoL and psychosocial health-related QoL. It is recommended that future studies perform longitudinal data collection to achieve a better understanding of the relationships of food addiction with psychosocial correlates and QoL. Finally, our findings provide preliminary evidence for the factors contributing to food addiction development and its health outcomes. It is necessary to investigate these relationships with various variables in order to find solutions for food addiction, which may be helpful for future health programs and interventions for food addiction.

## Figures and Tables

**Table 1:** Characteristics of participants according to food addiction status.

	Total (*N* = 593)	Food Addiction (*N* = 41)	No Food Addiction (*N* = 552)	*p* Value
Age (mean ± SD)	15.0 ± 1.4	15.1 ± 1.2	15.0 ± 1.4	0.740 ^a^
Female (*N*, %)	308 (51.9)	21 (51.2)	287 (52.0)	0.924 ^b^
BMI (mean ± SD)	19.6 ± 3.8	20.9 ± 4.0	19.5 ± 3.8	0.016 ^a^
Ethnicity (*N*, %)				
Han Chinese	497 (83.8)	35 (85.4)	462 (83.7)	0.779 ^b^
non-Han Chinese	96 (16.2)	6 (14.6)	90 (16.3)	
Single-child family (*N*, %)	449 (75.7)	30 (73.2)	419 (75.9)	0.694 ^b^
Intact family (*N*, %)	524 (88.4)	37 (90.2)	487 (88.2)	1.000 ^c^
Maternal education level (*N*, %)				
Less than junior high school	143 (24.1)	11 (26.8)	132 (23.9)	0.831 ^b^
Senior high school	163 (27.5)	12 (29.3)	151 (27.4)	
More than university degree	287 (48.4)	18 (43.9)	269 (48.7)	
Paternal education level (*N*, %)				
Less than junior high school	129 (21.8)	10 (24.4)	119 (21.6)	0.659 ^b^
Senior high school	163 (27.5)	13 (31.7)	150 (27.2)	
More than university degree	301 (50.8)	18 (43.9)	283 (51.3)	
Monthly household income (*N*, %)				
Less than 5000 yuan	132 (22.3)	11 (26.8)	121 (21.9)	0.797 ^b^
Between 5000 and 10,000 yuan	210 (35.4)	15 (36.6)	195 (35.3)	
Between 10,000 and 15,000 yuan	117 (19.7)	6 (14.6)	111 (20.1)	
More than 15,000 yuan	134 (22.6)	9 (22.0)	125 (22.6)	
Alcohol history (*N*, %)	12 (2.0)	2 (4.9)	10 (1.8)	0.199 ^c^
Depression (*N*, %)				
Depressed	165 (27.8)	20 (48.8)	145 (26.3)	0.002 ^b^
Non-depressed	428 (72.2)	21 (51.2)	407 (73.7)	
Self-esteem level (*N*, %)				
Low self-esteem	90 (15.2)	13 (31.7)	77 (13.9)	
Normal self-esteem	503 (84.8)	28 (68.3)	475 (86.1)	0.002 ^b^
Loneliness (*N*, %)				
Yes	114 (19.2)	14 (34.1)	100 (18.1)	0.012 ^b^
No	479 (80.8)	27 (65.9)	452 (81.9)	
Psychological problems (mean ± SD)				
Total difficulties	9.2 ± 5.8	13.2 ± 6.8	8.9 ± 5.6	<0.001 ^a^
Emotional symptoms	2.1 ± 2.2	3.2 ± 2.4	2.0 ± 2.1	<0.001 ^a^
Conduct problems	2.0 ± 1.7	2.9 ± 2.1	1.9 ± 1.6	<0.001 ^a^
Hyperactivity/inattention	2.8 ± 2.1	3.9 ± 2.1	2.7 ± 2.0	<0.001 ^a^
Peer relationship problems	2.4 ± 1.8	3.2 ± 2.4	2.3 ± 1.7	0.001 ^a^
Prosocial behavior	7.5 ± 2.2	7.6 ± 2.0	7.5 ± 2.2	0.800 ^a^
Quality of life total score (mean ± SD)	85.5 ± 12.6	76.0 ± 14.4	86.2 ± 12.2	<0.001 ^a^
Physical health (physical functioning)	87.2 ± 13.5	81.6 ± 16.0	87.6 ± 13.2	0.006 ^a^
Psychosocial health	84.5 ± 14.1	72.9 ± 15.7	85.4 ± 13.6	<0.001 ^a^
Emotional functioning	83.9 ± 20.0	67.4 ± 26.6	85.1 ± 18.9	<0.001 ^a^
Social functioning	89.6 ± 15.2	79.5 ± 21.4	90.4 ± 14.4	<0.001 ^a^
School functioning	80.2 ± 16.7	71.8 ± 16.3	80.8 ± 16.6	<0.001 ^a^

*p* value < 0.05 is statistically significant; ^a^ Two-tail Student *t*-test; ^b^ Pearson’s chi-square test; ^c^ Fisher’s Exact test.

**Table 2:** Psychosocial correlations of food addiction.

	Total *N* (%)	COR (95%CI) ^a^	AOR (95%CI) ^b^
Depression			
Non-depressed	428 (72.2)	1 (Reference)	1 (Reference)
Depressed	165 (27.8)	2.67 (1.41, 5.08) **	2.58 (1.32, 5.05) **
Self-esteem level			
Normal self-esteem	503 (84.8)	1 (Reference)	1 (Reference)
Low self-esteem	90 (15.2)	2.86 (1.42, 5.77) **	2.75 (1.31, 5.78) **
Loneliness			
No	479 (80.8)	1 (Reference)	1 (Reference)
Yes	114 (19.2)	2.34 (1.19, 4.63) *	2.30 (1.14, 4.65) *
Psychological problems			
Total difficulties	-	1.11 (1.06, 1.16) ***	1.11 (1.06, 1.17) **
Emotional symptoms	-	1.22 (1.08, 1.38) **	1.24 (1.09, 1.41) **
Conduct problems	-	1.34 (1,14, 1.58) ***	1.33 (1.13, 1.58) **
Hyperactivity/inattention	-	1.31 (1.13, 1.52) **	1.29 (1.11, 1.51) **
Peer relationship problems	-	1.29 (1.10, 1.52) **	1.31 (1.11, 1.55) **
Prosocial behavior	-	1.02 (0.88, 1.18)	1.03 (0.88, 1.20)

^a^ Simple logistic regression model. ^b^ Multivariable logistic regression model. COR: crude odds ratio, CI: confidence interval, AOR = adjusted odds ratio, odds ratio adjusted for age, gender, BMI, ethnicity, single-child family, intact family, maternal education level, paternal education level, monthly household income, and alcohol. Total difficulties and respective subscales of psychological problems were regarded as continuous variables in the model input. * *p* < 0.05, ** *p* < 0.01, *** *p* < 0.001.

**Table 3:** Associations of food addiction and quality of life.

	Model 1		Model 2		Model 3	
	β	95% CI	β	95% CI	β	95% CI
Total score	−10.22 ***	−14.15, −6.28	−9.65 ***	−13.60, −5.70	−6.40 ***	−9.83, −2.97
Physical health	−5.97 **	−10.22, −1.72	−5.30 *	−9.54, −1.05	−2.91	−6.93, 1.11
Psychosocial health	−12.48 ***	−16.84, −8.12	−11.97 ***	−16.37, −7.57	−8.27 ***	−12.05, −4.48
Emotional functioning	−17.63 ***	−23.84, −11.43	−17.23 ***	−23.50, −10.96	−12.14 ***	−17.51, −6.76
Social functioning	−10.86 ***	−15.62, −6.09	−10.74 ***	−15.55, −5.93	−7.07 **	−11.35, −2.78
School functioning	−8.96 **	−14.23, −3.68	−7.95 **	−13.24, −2.67	−5.59 *	−10.74, −0.45

β: regression coefficient, CI: confidence interval, Model 1: no adjustments, Model 2: adjusted for age, gender, BMI, ethnicity, single-child family, intact family, maternal education level, paternal education level, monthly household income, and alcohol, Model 3: additionally adjusted for depression, self-esteem level, and loneliness on the base of Model 2. * *p* < 0.05, ** *p* < 0.01, *** *p* < 0.001.
